# Kinetic investigation of *para*-nitrophenol reduction with photodeposited platinum nanoparticles onto tunicate cellulose

**DOI:** 10.1039/d2ra05507d

**Published:** 2022-10-28

**Authors:** T. A. Thiel, X. Zhang, B. Radhakrishnan, R. van de Krol, F. F. Abdi, M. Schroeter, R. Schomäcker, M. Schwarze

**Affiliations:** Technische Universität Berlin, Department of Chemistry TC8, Straße des 17. Juni 124 10623 Berlin Germany ms@chem.tu-berlin.de; Leibniz Institute for Catalysis Albert-Einstein-Straße 29a 18059 Rostock Germany; Institute for Solar Fuels, Helmholtz-Zentrum Berlin für Materialien und Energie GmbH Hahn-Meitner-Platz 1 14109 Berlin Germany; Institute for Active Polymers, Helmholtz-Zentrum Hereon Kantstrasse 55 14513 Teltow Germany

## Abstract

Photodeposition is a specific method for depositing metallic co-catalysts onto photocatalysts and was applied for immobilizing platinum nanoparticles onto cellulose, a photocatalytically inactive biopolymer. The obtained Pt@cellulose catalysts show narrow and well-dispersed nanoparticles with average sizes between 2 and 5 nm, whereby loading, size and distribution depend on the preparation conditions. The catalysts were investigated for the hydrogenation of *para*-nitrophenol *via* transfer hydrogenation using sodium borohydride as the hydrogen source, and the reaction rate constant was determined using the pseudo-first-order reaction rate law. The Pt@cellulose catalysts are catalytically active with rate constant values *k* from 0.09 × 10^−3^ to 0.43 × 10^−3^ min^−1^, which were higher than the rate constant of a commercial Pt@Al_2_O_3_ catalyst (*k* = 0.09 × 10^−3^ min^−1^). Additionally, the Pt@cellulose catalyst can be used for electrochemical hydrogenation of *para*-nitrophenol where the hydrogen is electrocatalytically formed. The electrochemical hydrogenation is faster compared to the transfer hydrogenation (*k* = 0.11 min^−1^).

## Abstract 2

Modified cellulose (ModCe) was used in a photodeposition process as a support material for platinum nanoparticles. The supported catalysts were investigated for the transfer hydrogenation of *para*-nitrophenol (PNP) to *para*-aminophenol (PAP).

## Graphical abstract

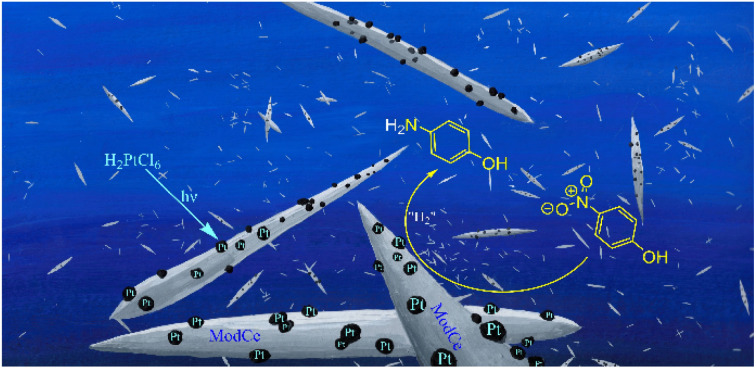

## 1. Introduction

Green chemistry has become increasingly important in overcoming environmental problems and climate change in the recent 20 years. It is defined by twelve principles, which include waste prevention, atom economy, less hazardous synthesis pathways, the design of benign chemicals, green solvents and auxiliaries, energy efficiency, renewable feedstocks, reducing derivatives, catalysis, degradability, real-time analysis for pollution prevention and overall accident prevention.^1^ Catalysis plays a key role in green chemistry, with a focus on reactions at high selectivity under mild reaction conditions. The applied catalyst can be in the form of catalyst complexes, metal nanoparticles, or bulk powders. In addition to the catalyst's activity, the catalyst separation from the reaction media is an important issue that can be solved by catalyst immobilization. Homogeneous catalyst complexes can be immobilized in a liquid–liquid two-phase system, as shown in the Ruhrchemie/Rhône-Poulenc process for the hydroformylation of propene to *n*-butyraldehyde.^2^ Nanoparticles can be immobilized in different ways. One approach uses stabilizers like micelles^3^ or polymers.^4,5^ The most common approach is the immobilization onto inert solid supports, which facilitates recycling but also avoids the agglomeration of nanoparticles. For example, gold nanoparticles can be deposited on various metal oxides like silica, cerium oxide, or zirconium oxide, using chloroauric acid (HAuCl_4_) as the Au precursor.^6^ Metal oxides are preferred as support materials because of their high chemical and thermal stability.^6,7^ However, since many reactions such as coupling reactions or hydrogenations of phenol^8^ or allylbenzene^9^ take place at temperatures below 150 °C or even at room temperature, an alternative is to use sustainable biomaterial supports like chitosan, starch, and cellulose.^6,10,11^ Cellulose is the most abundant natural polymer. In recent years, its use as a support material for metal nanoparticles has been investigated, as shown in the review of Kaushik and Moores.^12^ Cellulose nanoparticles such as nanocrystals and nanofibres can be obtained from the hydrolysis of cellulose with sulfuric acid, removing the amorphous regions. Conventional deposition methods of metal nanoparticles from metal salt precursors like impregnation, including calcination at high temperatures, are not suitable because cellulose degrades at temperatures above 170 °C. Therefore, preparing supported metal nanoparticles from an appropriate precursor is common using reducing agents. Salt precursors of various metals are mostly reduced using sodium borohydride, ascorbic acid, or hydrogen as reducing agents.^12^ Precursors can also be reduced directly on the unmodified cellulose surface with hydroxy groups or modified nanoparticle surfaces with, *e.g.*, aldehyde or thiol groups.^12^ The effort for nanoparticle deposition onto unmodified cellulose nanoparticle surfaces depends on the element and its precursor. The deposition of gold and silver nanoparticles is not elaborate. Their precursors, chloroauric acid and silver nitrate, are reduced by heating at 80–120 °C.^13,14^ Reducing hexachloroplatinic acid by the surface hydroxy groups of cellulose is more challenging as an autoclave and supercritical carbon dioxide are required.^15^

The phenomenon of photodeposition describes the irradiation-induced reduction of a metal salt precursor in the presence of a reducing agent. It is also referred to as photoreduction and photochemical deposition. The photodeposition of metal nanoparticles is widely known when immobilizing them onto semiconductors such as titanium dioxide, tungsten oxide, zinc oxide, or cadmium sulfide, stating the necessity of the band gap for the deposition mechanism.^16^ This contribution and the literature show that the necessity of a semiconductor is not true for all metal salt precursors. Nanoparticles from dissolved hexachloroplatinic acid (H_2_PtCl_6_), chloroauric acid (HAuCl_4_), silver perchlorate (AgClO_4_) and the chelate complex copper acetylacetonate (Cu(acac)_2_) can be directly photochemically synthesized in a solution or dispersion with support. Alcohols like ethanol and methanol are frequently used for the deposition of silver and platinum nanoparticles.^17^ Since alcohols can be used as a reducing agent and solvent simultaneously, the photodeposition turns out to be a very simple and green method. This contribution investigates the photodeposition of platinum nanoparticles onto cellulose, which is a popular method for the *in situ* immobilization of metal nanoparticles as co-catalyst in photocatalytic water splitting,^18^ as catalysts for the hydrogen of *para*-nitrophenol (PNP). The hydrogenation of PNP to *para*-aminophenol (PAP) is a common reaction in the pharmaceutical industry, *e.g.*, for the synthesis of the painkiller paracetamol,^19^ the cough expectorant ambroxol,^20^ and the anti-cancer drug sorafenib.^21^ PNP can be hydrogenated with molecular hydrogen, but only at elevated pressures above 20 bar.^22^ Due to the high pressure and the possibility of the formation of explosive gas mixtures, safety measurements and trained personnel are required. Therefore, alternative hydrogenation reactions have been investigated, such as transfer, photocatalytic or electrocatalytic hydrogenation. The choice of the hydrogen donor depends on the reaction type. Sodium borohydride (NaBH_4_) is often used in transfer hydrogenations in the liquid phase for catalyst testing,^23^ alcohols like methanol and ethanol are applied in photocatalytic hydrogenations,^24^ and water is used in the electrocatalytic hydrogenation. To study the catalytic performance of the as-prepared cellulose-supported Pt catalysts, the transfer-hydrogenation of PNP with NaBH_4_ was selected as the model reaction, because the reaction progress can be easily followed by UV-vis spectrometry. Two catalysts with different loadings were prepared *via* the photodeposition method and characterized. The catalytic activity was determined under different operating conditions and evaluated based on the catalyst preparation. For comparison, a commercial alumina-supported Pt catalyst was selected, too. In addition, hydrogen from electrochemical water-splitting was used *in situ* for the reduction of PNP as proof of both an alternative hydrogenation pathway and the possibility to apply the cellulose-supported platinum catalyst in an electrochemical reaction system.

## 2. Experimental part

### 2.1 Chemicals

For the preparation of the cellulose modification, the unmodified cellulose (UnCe), isolated from *Styela Clava* (Otto-Van-den-Berg-Company), and sulfuric acid (H_2_SO_4_, 96 wt%, ≥99%, Roth) were used. For the deposition of platinum nanoparticles onto cellulose, hexachloroplatinic acid solution (H_2_PtCl_6_, 8 wt% in H_2_O, Sigma Aldrich) was used as the precursor. For hydrogenation experiments, *para*-nitrophenol (PNP, ≥99%, Roth), sodium borohydride (NaBH_4_, 98%, Sigma Aldrich), sodium hydroxide (NaOH, 99%, Roth), and platinum on alumina (Pt@Al_2_O_3_, 1 wt%, Alfa Aesar) were used. The platinum ICP standard (1000 mg L^−1^, Sigma Aldrich), hydrogen chloride (HCl, 37 wt%, 99%, Roth), and nitric acid (HNO_3_, 68 wt%, 99%, Roth) were used for sample preparation and analysis. All chemicals were used without further modification.

### 2.2 Isolation of cellulose nanomaterials

The synthesis of modified cellulose (ModCe) by sulfuric acid hydrolysis was conducted by a method reported by van den Berg *et al.* with slight modifications.^25^ Under vigorous stirring, concentrated H_2_SO_4_ (120 mL) was slowly added to UnCe (4.5 g) and dispersed in water (120 mL) at 4 °C in a double-walled glass reactor (maximum volume 600 mL). The temperature was controlled by a thermostat (F6 C25, Haake). The temperature was increased to 60 °C, and the solution was stirred for 2 hours before it was cooled down again to 4 °C. The obtained suspension was filtered and washed over a small-pore glass fritted filter until the pH became neutral. The residue was freeze-dried (Alpha 1–4, Christ) for three days. It should be mentioned that the treatment of cellulose is a sensitive process and the result depends strongly on the experimental conditions (temperature, time, sulphuric acid concentration). The degree of functionalization is <1%, but this is sufficient to make the cellulose more hydrophilic and better dispersible.

### 2.3 Photodeposition of platinum nanoparticles

Two Pt@ModCe catalysts were prepared *via* photodeposition (PD) and are referred to as PDPt1 and PDPt2. For the photodeposition of platinum nanoparticles, H_2_PtCl_6_ (843 mg for PDPt1 and 131 mg for PDPt2) was placed in a round bottom flask. The ModCe dispersion (2.5 g L^−1^, 200 mL, *V*_MeOH_/*V*_water_ = 70/30) was added, and the mixture was purged with nitrogen for 15 min. The dispersions were irradiated by a 300 W Xe lamp (Quantum Design Europe) with the following conditions: no filter (*i.e.* full spectra) for 30 min in case PDPt1 and with 395 nm long-pass cut-off filter and for 105 min in the case PDPt2. The distance between the lamp and the flask was always 10 cm. The prepared catalysts were filtered, washed with water, and freeze-dried.

### 2.4 Catalyst characterization

#### 2.4.1 ICP-OES

Pt loadings of Pt@ModCe catalysts were determined from inductively coupled plasma atomic emission spectroscopy (ICP-OES) using a Varian 715 ES spectrometer (Agilent Technologies). The setup calibration was done with platinum standard solutions with concentrations of 2.5 ppm, 5 ppm, 10 ppm, 20 ppm, and 25 ppm. The nanoparticles from the catalysts were dissolved overnight by adding a mixture of HCl, HNO_3_, and H_2_SO_4_ (10 mL, *V*/*V*/*V* = 3/1/1) to the sample (20 mg). After this time, ModCe was filtered off, and the solution was diluted with water up to 50 mL. 5 mL of this solution were taken and further diluted up to 15 mL. The catalyst loading was calculated from the prepared sample solution's measured platinum concentration. Catalysts used in transfer-hydrogenation reactions were filtered and washed before ICP-OES measurements.

#### 2.4.2 TEM and diffraction pattern

To investigate the morphology, size distribution, and crystallinity of the Pt@ModCe catalysts, recordings using transmittance electron microscopy (TEM) with the TECNAI G220 (FEI company, USA, operated at 200 kV, with LaB6 electron emitter) were taken. The TECNAI device did direct recordings of diffraction patterns. The size distributions of platinum nanoparticles were generated by measuring the nanoparticle size in the TEM images using the image processing and acquisition software Digital Micrograph from Gatan Inc. (Version 3.43.3213.0). The data analysis and visualization program QTiPlot (Version 5.12.8) was used to fit the size distribution curves.

### 2.5 Hydrogenation of *para*-nitrophenol

The catalysts PDPt1 and PDPt2 were used to reduce *para*-nitrophenol with sodium borohydride as the hydrogen donor. The reaction was monitored with a UV-vis spectrometer (Lambda 35, PerkinElmer) with the method “Timedrive Lambda 35” (*λ* = 400 nm, time interval 2 s). Stock suspensions of the Pt@ModCe catalysts PDPt1 and PDPt2 (1 g L^−1^, *V*_MeOH_/*V*_water_ = 70/30), PNP (1 mM), and NaOH (4 g L^−1^ in MeOH and H_2_O) were prepared.

The reaction solution was stirred with a mixed drive (2mag cuvetteMIXdrive) and mix control (2mag MIXcontrol eco Control Unit) unit from 2mg-USA. NaBH_4_ (115 mg) was placed into a UV-vis cuvette for a standard reaction. Next, the aqueous PNP solution (100 μL) and an aqueous MeOH solution (*V*_MeOH_/*V*_water_ = 70/30) was added and stirred until the NaBH_4_ was dissolved. The measurement program was started, and the reaction was initiated by adding the Pt@ModCe dispersion (0.25 mL of PDPt, 1.0 mL of PDPt2). The MeOH fraction and the catalyst amount were varied, and each experiment was conducted three times. A pseudo-first-order reaction law eqn (1) was assumed to evaluate the reaction performance, where *c*_PNP_ is the concentration of PNP, *t* the time, and *k* is the reaction rate constant. The linearized form (eqn (2)) was used to obtain the reaction rate constant.1
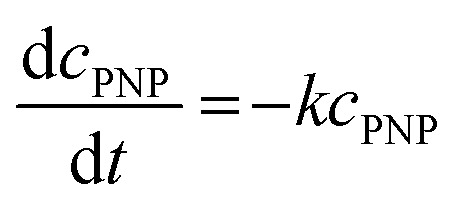
2
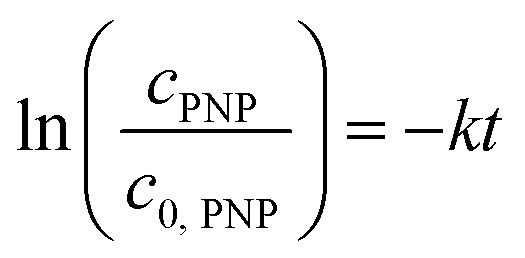


The absorption intensity *I* of the solution is proportional to the concentration of PNP for low concentrated solutions where the Lambert–Beer law is applicable. Eqn (2) can thus be written as:3
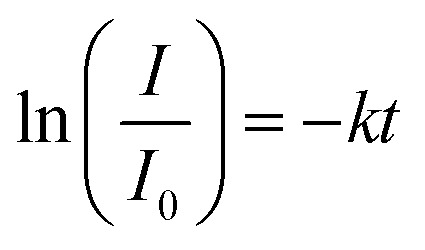


The activity *A* of the catalyst was calculated as follows:4
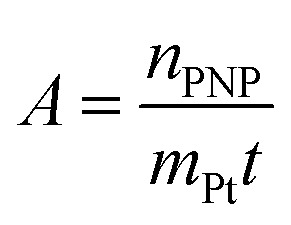


The *p*-nitrophenol amount is *n*_PNP_, *m*_Pt_ is the mass of platinum nanoparticles, and *t* is the time. The reaction rate constant and catalyst activity were determined for a reaction time of 400 s.

Electrochemical measurements were performed in a commercial Micro Flow Cell (ElectroCell) under three-electrode configuration using a VersaSTAT 3 potentiostat/galvanostat (AMETEK). Uncompensated resistance was obtained from impedance measurements, and iR corrections were performed to the applied voltage. A Pt cathode (Pt foil, 0.05 mm thick, Premion®, 99.99%, Thermo Scientific) was used as the working electrode with a geometric active area of 10 cm^2^. The counter electrode was a dimensionally stable anode (DSA®), and the reference electrode was a leak-free Ag/AgCl (3.4 M KCl) electrode (LF-1, Innovative Instruments Ltd). A cation exchange membrane (NRE-212, Nafion™, thickness 0.002 inches) was placed between the anolyte and the catholyte chambers. The anolyte was 1 M potassium phosphate (KP_i_) buffer solution (pH = 7), and the catholyte was either 1 M KP_i_ with added 0.03 mM of *p*-nitrophenol (1 M KP_i_ + PNP) or 1 M KP_i_ with 0.03 mM of *p*-nitrophenol and 0.08 g L^−1^ of PDPt1 catalyst (1 M KP_i_ + PNP + PDPt1). Both the anolyte and catholyte solutions (100 mL) were continuously degassed with Ar during the electrochemical measurements to prevent oxygen contamination. The solutions were circulated by two peristaltic pumps (TBE/200, MDX Biotechnik International GmbH) with a flow rate of 120 mL min^−1^. Galvanostatic measurements were performed at a current density of −2 mA cm^−2^ for 2 hours. 1 mL of the electrolyte were collected periodically through a septum located right after the outlet of the catholyte, and the progress of the hydrogenation reaction was monitored by UV-vis spectroscopy (*vide supra*).

The 1 M KP_i_ buffer solutions were prepared used KH_2_PO_4_ (≥99.0%, Sigma-Aldrich) and K_2_HPO_4_·3H_2_O (≥99.0%, Sigma-Aldrich). The water used in all experiments was obtained from a Milli-Q Integral system with a resistivity of 18.2 MΩ cm.

## 3. Results and discussion

### 3.1 Catalyst preparation and characterization

**Fig. 1:**
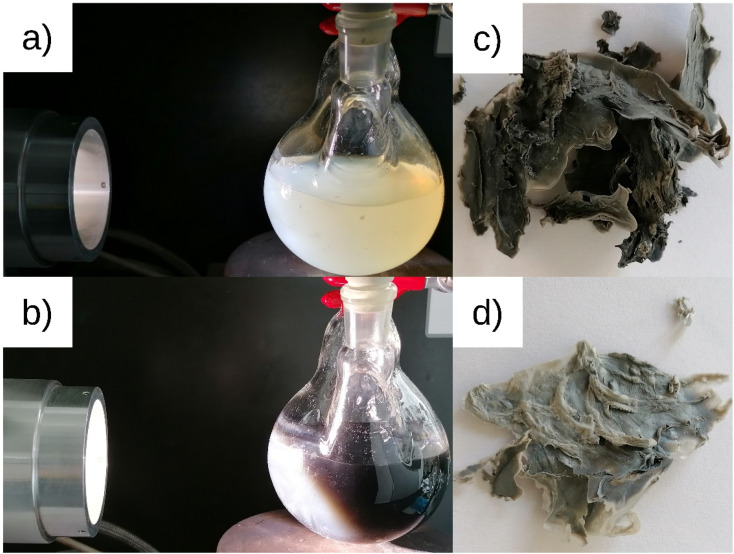
ModCe dispersion with precursor at the beginning (a) and end (b) of platinum nanoparticles photodeposition process leading to the Pt@ModCe catalysts PDPt1 (c) and PDPt2 (d).

The catalysts were characterized by their loadings, nanoparticle sizes and size distributions, and crystallinity. The values are listed in Tables 1 and 2. Fig. 1 shows the catalyst preparation with photodeposited platinum nanoparticles on cellulose and the resulting catalysts PDPt1 and PDPt2. The precursor suspension (Fig. 1a) has a pale-yellow color, which turns to dark grey (Fig. 1b), indicating the formation of platinum particles. The determined platinum loadings are 2.7% for PDPt1 and 0.8% for PDPt2, with corresponding deposition yields of 42% and 80%, respectively. The difference in deposition yields probably originates from different irradiation periods (30 min *vs.* 105 min). However, it is also known that support properties and the interaction between support and nanoparticles are important for the deposition process. Schröder *et al.* used the photodepostion method to immobilize Pt nanoparticles onto carbon nitride, and the deposition yields were below 10%.^26,27^ The support effect in the deposition of metal nanoparticles was studied by Parapat *et al.*^28^ using a colloidal deposition method, showing that with an appropriate support, high deposition yields can be obtained. Here, the loading is relatively high (>40%), showing that cellulose can be used as a support material for the deposition of Pt nanoparticles. Further, the higher deposition yield for PDPt2 can be explained by using a cut-off filter. When using the full spectrum for the photodeposition process, the reduction of the platinum salt is fast. It produces many nanoparticles in the liquid phase, having less time to deposit onto the cellulose surface. In contrast, the reduction is much slower with the filter, so the deposition is more efficient.

**Table 1:** Overview of the average particle sizes *d*_NP_, the variance of the particle size *σ*, theoretical loading, loadings determined by ICP-OES, and the resulting deposition yield

Catalyst	*t*_irradiation_ (min)	Cut-off filter (nm)	*d*_NP_ (nm)	*σ* (nm)	Theoretical loading (wt%)	Loading (wt%)	Deposition yield (%)
PDPt1	30	None	4.6	0.9	6.4	2.7	42
PDPt2	105	395	2.3	0.9	1.0	0.8	80
Pt@Al_2_O_3_	—	—	5.8	1.3	1.0^a^	—	—

a Catalyst loading according to the product sheet.

**Table 2:** Overview of the measured reciprocal lattice value 1/*r*, the calculated lattice plane distance *d*, and the determined Miller indices *hkl* for the platinum catalysts

Catalyst	1/*r* (nm^−1^)	*d* (Å)	Δ*d* (Å)	*hkl*
PDPt1	4.4	2.3	0.1	(111)
	5.1	2.0	0.1	(200)
	7.2	1.4	0.1	(220)
PDPt2	4.5	2.2	0.1	(111)
	5.2	1.9	0.1	(200)
	7.2	1.4	0.1	(220)
Pt@Al_2_O_3_	4.4	2.3	0.1	(111)
	5.1	2.0	0.1	(200)
	7.3	1.4	0.1	(220)

**Fig. 2:**
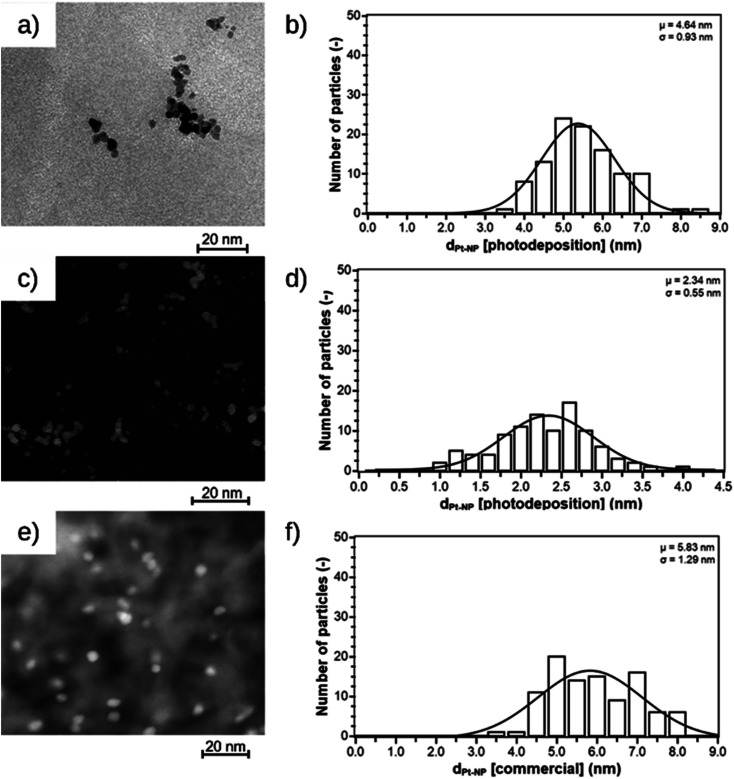
TEM images and size distribution of nanoparticles of PDPt1 (a and b), PDPt2 (c and d), and the commercially available Pt@Al_2_O_3_ catalyst (e and f).

**Fig. 3:**
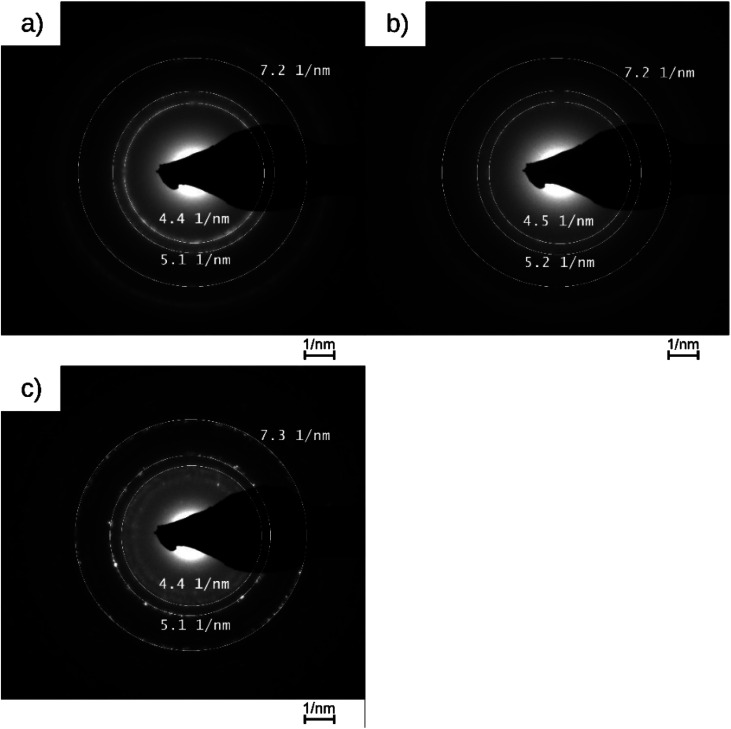
Diffraction patterns of PDPt1, PDPt2, and Pt@Al_2_O_3_ with the measured reciprocal lattice value.

The Pt@ModCe catalysts were characterized by TEM (Fig. 2a, c, and e), showing round-shaped nanoparticles of PDPt1, PDPt2, and Pt@Al_2_O_3_, and the particle sizes and size distributions were obtained from these images (Fig. 2b, d, and f). PDPt2 shows a smaller average particle size of 2.3 nm than PDPt1 with 4.6 nm. The difference in average particle size originates from the precursor concentration (128 μg L^−1^ for PDPt2 and 822 μg L^−1^ for PDPt2), which was also shown in earlier publications.^16,29^ The particle size variation of PDPt1 and PDPt2 is with a value of 0.9 nm, identical and independent from the precursor concentration. The average particle size of platinum particles of the commercial catalyst Pt@Al_2_O_3_ is 5.8 nm, significantly larger than the PDPt2 and slightly larger than the PDPt1 particles. The particle size variation of 1.3 nm is slightly larger than that of PDPt1 and PDPt2. The crystallinity of platinum nanoparticles was investigated by determining the Miller indices of the lattice planes through the diffraction patterns recorded for PDPt1, PDPt2, and Pt@Al_2_O_3,_ as shown in Fig. 3. The electron diffraction of polycrystalline materials leads to diffraction patterns with sharp rings. The reciprocal lattice value 1/*r* is the radius of these rings and was measured to calculate the plane distance *d*. The lattice plane distance is the characteristic value for each crystal composition's lattice plane and was determined by the internal database of the TEM devices (JCPDS 46-1043 and 04-0802). The values 1/*r*, *d*, and Miller indices are listed in Table 2. For all platinum crystals, at least the two Miller indices (111) and (200), and sometimes additionally (220), were identified. In the diffraction patterns of Pt@Al_2_O_3_, the aluminum oxide reflexes can overlay platinum's reflexes because the lattice plane distances are similar. However, since no other typical reflexes of platinum are found, the same lattice plane distances can be assumed. The combination of the Miller indices of the lattice planes indicates a face-centered cubic lattice structure (fcc) of platinum nanocrystals, which is also the characteristic lattice structure of platinum.^30,31^ Consequently, the deposition method does not influence the crystallinity when platinum nanoparticles are formed. The photodeposition of platinum nanoparticles on a non-semiconductor like cellulose provides nanoparticles with platinum-characteristic fcc lattice structure in small, narrow distributed sizes. Best deposition yield and smallest nanoparticles were obtained with low precursor concentration.

### 3.2 Hydrogenation of PNP

#### 3.2.1 Reaction progress and data evaluation

**Fig. 4:**
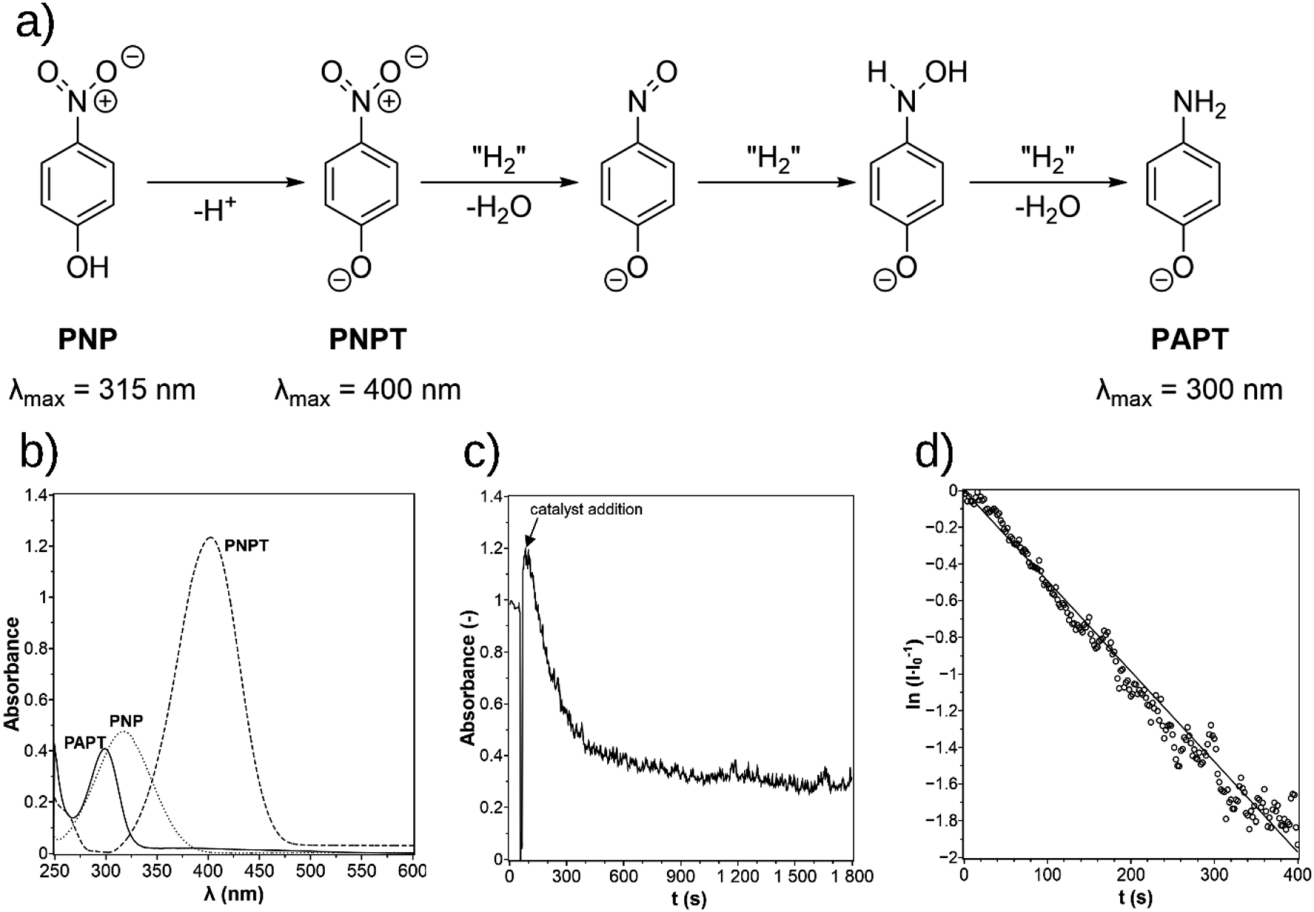
The mechanism for the reduction of *p*-nitrophenol (PNP) over *p*-nitrophenolate (PNPT) to *p*-aminophenolate (PAPT, a)^11^ and the UV-vis spectra of PNP, PNPT, and PAPT (b). The absorbance–time diagram of the experimental data (c) and the determination of the rate constant in the 
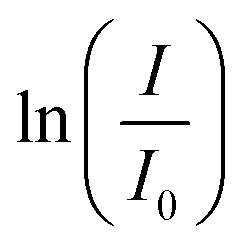
-time plot (d).

The *p*-nitrophenol (PNP) reduction to *p*-aminophenol (PAP) is frequently used as a model reaction to investigate heterogeneous catalysts whereby sodium borohydride is used as the hydrogen donor for the transfer hydrogenation reaction that proceeds in water at high pH values.^11,23^ This reaction was used for catalyst testing of the photodeposition-prepared PDPt1 and PDPt2 and the commercial Pt@Al_2_O_3_ catalyst as the reference. The reduction of the nitro group proceeds over two intermediates (Fig. 4a). First, the NaBH_4_ deprotonates the hydroxyl group of PNP to *p*-nitrophenolate (PNPT), causing the shift of its absorption maximum from 315 nm to 400 nm, as shown in Fig. 4b. That enables the monitoring of the reaction *via* UV-vis spectroscopy. Second, the nitro group is reduced to *p*-aminophenolate (PAPT) in three steps. In each step, two hydrogens (a hydride and a proton) are transferred.^11^ Finally, with the complete decomposition of NaBH_4_ and the neutralization of the solution, PAPT is protonated to PAP. The obtained data for an experiment was plotted as an absorbance–time diagram (Fig. 4c). The maximum of the curve occurs after the catalyst was added and was marked as time *t* = 0 for further evaluation with eqn (3). The absorbance at *t* = 0 was *I*_0_. The data was then plotted in an 
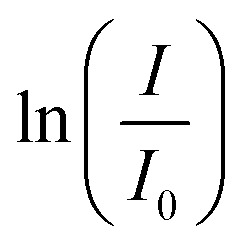
-time diagram (Fig. 4d). The slope of the regression line was the rate constant *k*.

#### 3.2.2 Impact of reaction solvent

**Fig. 5:**
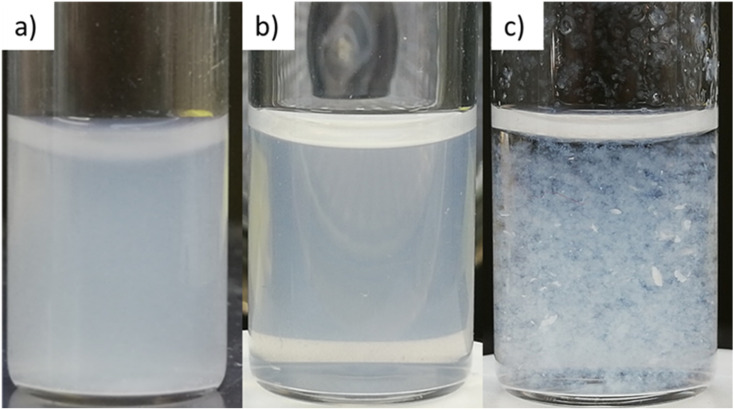
ModCe dispersions (1.5 mg L^−1^) in water (a), in a water–methanol mixture (V/V = 70/30) (b), and in methanol (c).

Unmodified cellulose can not be dispersed in polar solvents like water or methanol; therefore, it was modified by sulphuric acid treatment. After this modification, cellulose can be dispersed. However, the solvent still influences the homogeneity, as shown in Fig. 5. The best dispersion of cellulose is obtained for a mixture of methanol and water with a methanol content of about 70 vol% (Fig. 5b). For pure water (Fig. 5a), the dispersion is quite homogenous. However, in the case of pure methanol (Fig. 5c), an inhomogeneous dispersion is obtained.

**Fig. 6:**
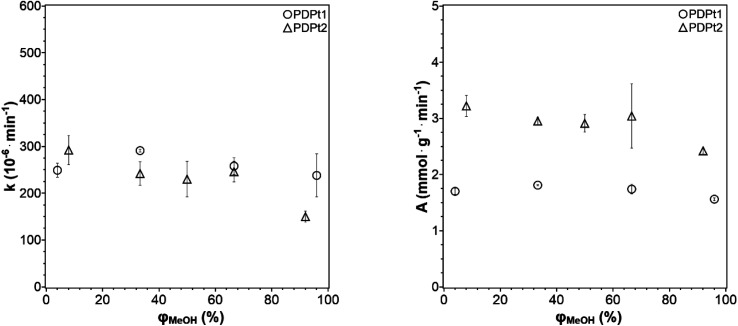
Reaction rate constant k (left) and catalytic activity A (right) as a function of the methanol volume fraction *φ*. Conditions: 250 μg PDPt1, 500 μg PDPt2, 33 μM PNP, *V* = 3 mL.

In the investigation of how the solvent influences the catalytic activity and to select the solvent system for the following experiments, the methanol fraction was varied in the range 4–96 vol%. The reaction rate constant *k* and the activity A were determined as described in Section 3.2.1 and plotted in Fig. 6 against the methanol volume fraction *φ*.

For the high-loaded PDPt1 (2.7 wt% Pt), the reaction rate constant and the activity do not change with variation of the methanol fraction. For the low loaded PDPt2 (0.8 wt% Pt), the reaction rate constant and the activity are slightly lower for a methanol fraction of 92 vol%. The latter is probably due to the limitation of mass transport caused by the swelled cellulose, increasing the viscosity of the solution. The platinum concentrations with 11 μM for PDPt1 and 3 μM for PDPt2 for the reduction of 33 μM PNP are considerably high, providing sufficient activity centers to lower or, in the case of PDPt1, avoid the impact of the cellulose swelling on the reaction rate. However, the overall impact of cellulose swelling with increasing methanol fraction on reaction rate and activity is negligible. The activity values of PDPt2 are generally higher than those of PDPt1 because of the lower loading of PDPt2 and, therefore, lower Pt concentration. The mean activity of PDPt2 is about 3 mmol g^−1^ min^−1^ which is about 1.7 times higher than the activity of PDPt1. The comparison of the platinum mass ratio *m*(PDPt2)/*m*(PDPt1) of 0.7 with the catalysts activity ratio *A*(PDPt2)/*A*(PDPt1) of 1.7 shows that the activity is not influenced by the catalyst mass exclusively. If that is the case, both values would be identical. The nanoparticle surface ratio (NSR) was calculated from eqn (5):5
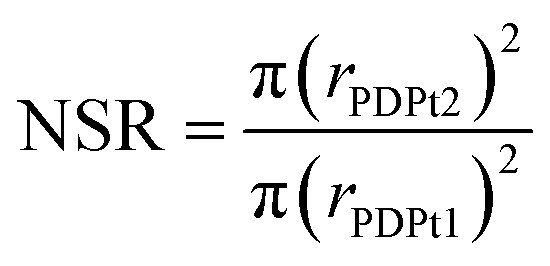


The NSR is 0.3, which is close to the value of the ratio of the activity ratio and mass ratio, indicating that the catalyst activity is mainly influenced by the particle size of the active catalytic species.

#### 3.2.3 Impact of catalyst concentration

**Fig. 7:**
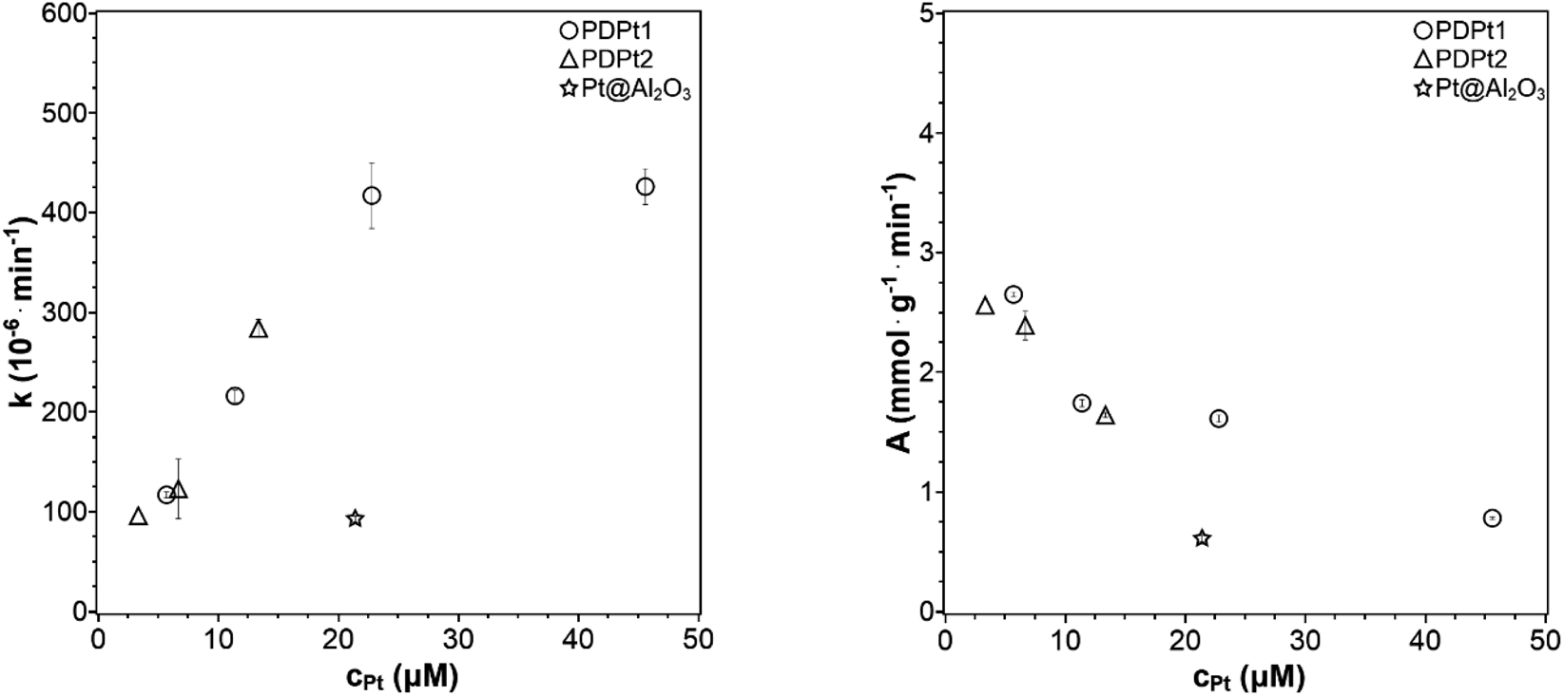
Reaction rate constant *k* and activity *A* plotted against the concentration of platinum nanoparticles (33 μM PNP, *V*_MeOH_/*V*_water_ = 70/30).

For the variation of the catalyst concentration, the added volumes of the catalyst stock suspension were variated to be 125 μL, 250 μL, 500 μL, and 1000 μL for PDPt1 as well as 250 μL, 500 μL, and 1000 μL for PDPt2. The catalyst concentration is given as the concentration of platinum nanoparticles calculated from the added catalyst amount multiplied by the platinum loading measured by ICP-OES. The obtained rate constants and activities are plotted against the platinum concentration in Fig. 7. The reaction rate constant and the activity of a commercial Pt@Al_2_O_3_ catalyst is shown as a reference. For the same platinum concentration, the reaction is four-fold faster for the cellulose-supported catalysts prepared *via* photodeposition. With increasing catalyst concentration, the reaction constant increases first but will become constant later. PDPt1 indicates a saturation curve with a maximum *k* value of 0.43 × 10^−3^ min^−1^ for platinum concentrations higher or equal to 23 mM, caused by mass transport limitation at the solid–liquid interface. The PNP to PAP conversion is faster than the molecular diffusion of the reactants onto and from the catalyst. Independently of how many active centers are available, the diffusion rate of the molecules remains the same and prevents the increase of the reaction rate. The saturation level of PDPt2 is not reached within the same concentration range because of the low loading of PDPt1. Due to the low platinum concentration in the stock suspension, the required suspension volume would have been too large for the cuvette in further experiments. The activity decreases with increasing platinum concentration and decreases slightly more for PDPt2 than for PDPt1.

No reference was found in the literature where supported platinum nanoparticles catalyze the PNP reduction with the same concentrations of platinum and PNP as used here. One example was found by König *et al.*^9^ with 32 μM PNP in a reaction volume of 3 mL. They studied a catalyst with ethoxylated polyethylene imine as support with a Pt loading of 30 wt%. Thus, the platinum concentration was 513 μM resulting in a rate constant of 0.165 min^−1^ and an activity of 63 μmol g^−1^ min^−1^. Further discussions with these reported catalysts are only possible with additional characterizations like particle size. Another example is the publication of Lin *et al.*^32^ with 13 μM PNP in a reaction volume of 3 mL and a catalyst with wood cellulose as support (0.1 wt%, Pt). The size of Pt particles was 2.3 nm, which is similar to the particle size of PDPt2 and smaller than the particle size of PDPt1. Thus, the platinum concentration was 0.9 μM resulting in an activity of 0.6 mmol g^−1^ min^−1^, which is a lower activity than obtained for Pt catalysts in this work despite the low loading and small Pt nanoparticles. These results show that the Pt@ModCe catalysts are active for PNP transfer-hydrogenation. The activities are in the same order of magnitude or even better than the reported ones.

#### 3.2.4 Electrocatalytic hydrogenation of *para*-nitrophenol

The possibility to utilize electrochemically generated hydrogen to hydrogenate PNP to PAP using the Pt@ModCe catalyst PDPt1 was also explored. Three-electrode electrochemical configuration was used, with a Pt cathode as the working electrode, a dimensionally stable anode as the counter electrode, and an Ag/AgCl electrode as the reference electrode. 1 M potassium phosphate (KP_i_, pH 7) buffer was used as the anolyte, and the catholyte was 1 M KP_i_ and 0.03 mM PNP without and with PDPt1 catalyst (80 mg L^−1^). Chronopotentiometry measurement was performed, and liquid samples were then collected from the catholyte at various times to detect the progress of hydrogenation of PNP to PAP by measuring the normalized absorption intensity (*I*/*I*_0_) as described above.

**Fig. 8:**
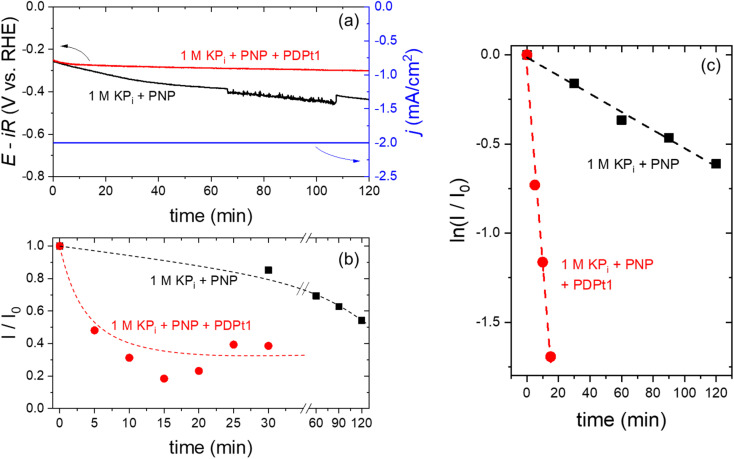
Chronopotentiometry (−2 mA cm^−2^, 1 M KP_i_ buffer as catholyte, Pt foil cathode) with PNP (black) only and with PNP and PDPt1 (red). The applied current density is shown as a blue curve (a). Plotted normalized absorption intensity (*I*/*I*_0_) against time (b) and 
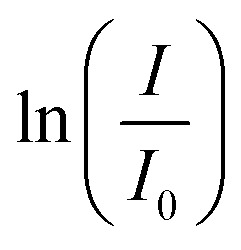
-time plot obtained from samples of the catholyte solution during the electrochemical experiment in 1 M KP_i_ buffer with only PNP (black) and 1 M KP_i_ buffer with PNP and PDPt1 (red) (c). The dashed lines in (b) are guides to the eye, and the dashed lines in (c) are linear fits of the data points.

At a constant current density of −2 mA cm^−2^, the chronopotentiometry curves are shown in Fig. 8a. In the absence of PDPt1 in the catholyte (black curve), the measured potential gradually decreases to more negative values. *I*/*I*_0_ continuously decreases as a function of time (Fig. 8b – black curve) within the 2 hour measurement period, indicating a decrease in the concentration of PNP and an increase in the concentration of PAP. Since no hydrogenation catalyst is added, the electrochemical hydrogenation of PNP to PAP occurs directly on the surface of the Pt cathode. The instability of the measured potential is attributed to the transformation of Pt surface during the hydrogenation reaction, as reported previously during the electrochemical hydrogenation of maleic acid.^33^ This limitation is overcome when the PDPt1 hydrogenation catalyst is added to the catholyte; the measured potential remains stable within two hours of the electrochemical measurement (see Fig. 8a – red curve). In this configuration, hydrogenation of PNP occurs in a coupled mechanism, *i.e.*, H_2_ electrochemically evolves on the surface of the Pt cathode, which is subsequently used for the catalytic hydrogenation of PNP over the PDPt1 catalyst within the catholyte compartment. The coupled electrochemical hydrogenation results in a much more rapid initial decrease of *I*/*I*_0_, which then saturates at 0.30 ± 0.08 after ∼10 min (see Fig. 8b – red curve). This indicates that complete hydrogenation of PNP to PAP finishes within 10 min. Assuming that the faradaic efficiency for H_2_ is 100% on the Pt cathode, this corresponds to a H_2_-to-PAP conversion of ∼5%, *i.e.*, 5% of the electrochemically generated H_2_ are directly used *in situ* to hydrogenate PNP to PAP. Further improvement of the conversion is expected by optimizing the electrochemical cell parameters (*e.g.*, flow rate, electrolyte composition), but this is beyond the scope of the current study. Fig. 8c shows the natural logarithm of *I*/*I*_0_ plotted as a function of time. Based on eqn (3), the hydrogenation rate constant can be determined from the slope of the curves. The hydrogenation rate constant increases from 5 × 10^−3^ min^−1^ to 0.11 min^−1^ with the addition of PDPt1 into the catholyte. This 20-fold increase in rate constant highlights the important benefit of coupled *vs.* direct electrochemical hydrogenation. The same PNP and PDPt1 catalyst concentrations were used for coupled electrocatalytic hydrogenation and the previously shown transfer hydrogenation with NaBH_4_. The rate constant for the coupled hydrogenation is approximately 256-fold higher than the highest reaction rate of the classic model reaction (0.43 × 10^−3^ min^−1^), emphasizing the high effectivity of this reaction concept.

## 4. Conclusion

Platinum nanoparticles were deposited onto cellulose *via* photodeposition. Two catalysts were manufactured this way using no filter (PDPt1) and a 395 nm cut-off filter (PDPt2) during irradiation. PDPt1 and PDPt2 were compared to a commercial Pt@Al_2_O_3_ catalyst for characterization and testing with the catalytic transfer hydrogenation of PNP with NaBH_4_. The characterization by transmission electron microscopy showed that the platinum particles on cellulose are with 4.6 nm for PDPt1 and 2.3 nm for PDPt2 smaller and have a narrower size distribution than the particles on Pt@Al_2_O_3_ (5.8 nm). Not using a cut-off- filter probably leads to a lower deposition yield of PDPt1 (42%) than PDPt2 (80%). Platinum particles of all catalysts are round-shaped and have the same crystallinity. The testing with the transfer hydrogenation of PNP, evaluated with first-order reaction rate law, investigated the influence of the solvent composition and catalyst concentration. The dispersion becomes inhomogeneous, with increasing methanol volume fraction influencing the reaction rate constant negligibly. PDPt1 shows a higher conversion than PDPt2 due to its higher loading. However, the comparison of the activity of the manufactured catalysts showed that nanoparticle size mainly influences the activity of the catalysts. Varying the catalyst concentration, the transport limitations lead to a maximum possible rate constant of 0.43 × 10^−3^ min^−1^. The activity values of the prepared platinum nanoparticles are similar to or better than those of other reports. PDPt1 and PDPt2 are considerably more active than the commercial catalyst. PDPt1 was additionally tested for electrochemical hydrogenation of PNP. In this coupled reaction, the *in situ*, electrochemically generated molecular hydrogen is directly used for the PNP reduction. This hydrogenation method, forming molecular hydrogen at the platinum electrode and its subsequent consumption for the PNP hydrogenation, achieves a vastly higher reaction rate (256-fold) than the transfer hydrogenation method with sodium borohydride. This example of a coupled electrocatalytic hydrogenation shows the potential and necessity to continue exploring this technique.

## Conflicts of interest

The authors declare no conflicts of interest.

## Supplementary Material
